# Supplementary material for: Predicting visual function by interpreting a neuronal wiring diagram
Source: Nature. 2024 Oct 2;634(8032):113–23. doi: 10.1038/s41586-024-07953-5 (PMC11446822; doi:10.1038/s41586-024-07953-5)
Supplement: Supplementary file 6 — CRF and ERF predictions for individual TmY4 and TmY9 cells. Analogous to Supplementary Data 3, but for TmY target types. Shown are the top four monosynaptic pathways, the strongest pathway passing through each of the top ten intermediary types (ranking from Extended Data Fig. 7), and the trisynaptic pathway Tm1–TmY–Dm3–TmY (see the section entitled Prediction of spatial normalization). [file 41586_2024_7953_MOESM6_ESM.zip › DataS4/TmY9q/720575940627517266.pdf]

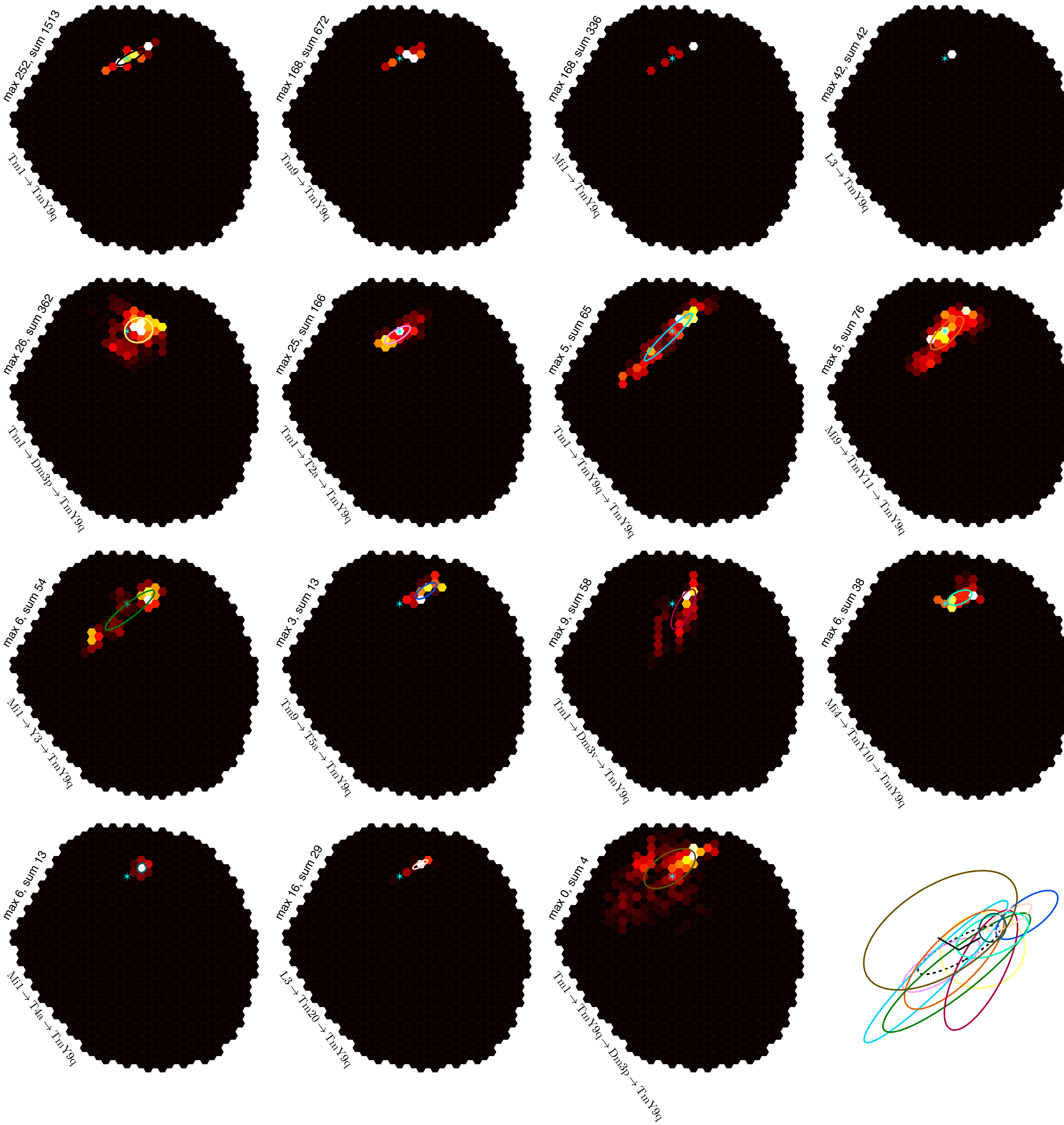
max 252, sum 1513
Tm1 → TmY9q
max 168, sum 672
Tm9 → TmY9q
max 168, sum 336
Mi1 → TmY9q
max 42, sum 42
L3 → TmY9q
max 26, sum 362
Tm1 → Dm3p → TmY9q
max 25, sum 166
Tm1 → T2a → TmY9q
max 5, sum 65
Tm1 → TmY9q → TmY9q
max 5, sum 76
Mi9 → TmY11 → TmY9q
max 6, sum 54
Mi1 → Y3 → TmY9q
max 3, sum 13
Tm9 → T5a → TmY9q
max 9, sum 58
Tm1 → Dm3v → TmY9q
max 6, sum 38
Mi4 → TmY10 → TmY9q
max 6, sum 13
Mi1 → T4a → TmY9q
max 16, sum 29
L3 → Tm20 → TmY9q
max 0, sum 4
Tm1 → TmY9q → Dm3p → TmY9q
